# A strong case for third-party testing

**DOI:** 10.7554/eLife.93329

**Published:** 2023-11-14

**Authors:** Fridtjof Lund-Johansen

**Affiliations:** 1 Department of Immunology, Oslo University Hospital Oslo Norway

**Keywords:** antibody, antibody validation, recombinant antibody, polyclonal antibody, antibody specificity, Human

## Abstract

A strategy to identify high-quality commercially available antibodies for research reveals extensive use of non-specific antibodies and offers solutions for future large-scale testing.

**Related research article** Ayoubi R, Ryan J, Biddle MS, Alshafie W, Fotouhi M, Bolivar SG, Moleon VR, Eckmann P, Worrall D, McDowell I, Southern K, Reintsch W, Durcan TM, Brown CM, Bandrowski A, Virk HS, Edwards AM, McPherson PS, Laflamme C. 2023. Scaling of an antibody validation procedure enables quantification of antibody performance in major research applications. *eLife* **12**:e91645. doi: 10.7554/eLife.91645.

Antibodies are used extensively by scientists to detect proteins in body fluids, tissue sections, cells and subcellular compartments. However, researchers often find that commercial antibodies either fail to recognize their target and/or bind to additional, non-target proteins (Baker, 2015; Marx, 2013). For example, a study published in 2013 showed that only 48% of 3,313 antibodies recommended for a technique called western blotting recognized their intended protein (Algenäs et al., 2014). Furthermore, a 2015 commentary stated that universities in the United States waste over $350 million annually by purchasing antibodies that do not work as advertised (Bradbury and Plückthun, 2015).

Although attempts have been made to establish standardized validation criteria, it was not clear if this had helped to improve the situation (Uhlen et al., 2016). Consequently, researchers often end up choosing antibodies based on how many times they have been cited in papers. Now, in eLife, Carl Laflamme from McGill University and colleagues – including Riham Ayoubi as first author – report results from a comprehensive third-party test of 614 commercial antibodies (Ayoubi et al., 2023).

The team (who are based at various institutes in the United States, Canada and the United Kingdom) studied antibodies for 65 neuroscience-related targets. This sample included monoclonal antibodies (derived from a single cell that has been cloned), polyclonal antibodies (derived from immune cells of an animal) and more recently developed recombinant antibodies (derived from synthetic genes).

Ayoubi et al. tested antibody specificity in cell lines that expressed high levels of mRNA for a target protein (positive control), and cells where the protein was absent due to the target gene being knocked out using the CRISPR Cas9 system (negative control). Testing included three widely utilized techniques for detecting the presence or distribution of proteins: western blots, which are the cornerstone of most manufacturers’ validation protocols; immunofluorescence microscopy, which visualizes protein location; and capturing proteins from cell lysates, an application that manufacturers rarely test for.

The experiments identified well-performing antibodies for 50 of the 65 proteins. On average, the recombinant antibodies performed better across the three tests, with only around a third of polyclonal and monoclonal antibodies recognizing their target in the experimental approaches they were recommended for.

Alarmingly, through a literature search, Ayoubi et al. found that the failing antibodies had been used in hundreds of studies, leaving little doubt that poor antibodies contribute to the reproducibility crisis in basic research. As a result of the study, 73 antibodies that failed to recognize their intended target have been discontinued and recommendations have been changed for another 153.

The findings of Ayoubi et al. suggest that recombinant antibodies are superior to monoclonal and polyclonal antibodies. However, there is large variation in how different manufacturers test their products. It is therefore possible that the small number of manufacturers responsible for making most of the recombinant antibodies tested may have higher standards of quality control, resulting in better performance. Manufacturers also tend to resell antibodies from a wide range of external suppliers, without in-house testing. It would be interesting to know how many of the monoclonal and polyclonal antibodies included in this study had been validated directly by the manufacturers that submitted them.

Going forward, Ayoubi et al. propose that grant institutions should fund centralized third-party validation in order to identify the most effective antibodies, especially as manufacturers cannot be expected to generate negative controls and re-test their entire inventory. There are, however, some limitations to such a centralized approach, as a single entity can only test a small fraction of the hundreds of thousands of available antibodies. How many antibodies should be tested to ensure that the best are not missed? Selecting the worthiest candidates could also be difficult: in some instances, the best antibody is one that has rarely been used in publications (Andersson et al., 2017).

It could also be argued that recombinant antibodies should be prioritized as they can be produced in large quantities indefinitely and were found to be superior. However, this approach could favor the few large manufacturers that already have good quality control processes in place, and harm manufacturers with well-validated monoclonal and polyclonal antibodies. The weak performance found in this study does not necessarily indicate that non-recombinant antibodies are inferior, but rather that those included in the study may have been poorly validated prior to submission.

Nevertheless, the findings of Ayoubi et al. present a compelling case for professional third-party testing that is independent from the manufacturer of the antibody and the eventual user of the product. The raw data has also been posted on the ZENODO open repository, which will be a highly useful resource for future studies. Providing third-party validation data that manufacturers can use in their own marketing materials will incentivize companies to submit their antibodies for testing, which several leading manufacturers have already done.

A strategy that could complement this approach would be to fund a comprehensive repository of knockout cells to use as negative controls. The resource could be made accessible to both industrial and academic institutions, effectively transforming laboratories across various sectors into potential testing sites. This initiative would facilitate testing of a far greater number of antibodies and provide researchers with much needed tools to verify their results prior to publication.
